# CRISPR/Cas-Based Approaches to Study Schizophrenia and Other Neurodevelopmental Disorders

**DOI:** 10.3390/ijms24010241

**Published:** 2022-12-23

**Authors:** Artemiy O. Kurishev, Dmitry S. Karpov, Nonna I. Nadolinskaia, Anna V. Goncharenko, Vera E. Golimbet

**Affiliations:** 1 Mental Health Research Center, Kashirskoe sh. 34, 115522 Moscow, Russia; 2 Center for Precision Genome Editing and Genetic Technologies for Biomedicine, Engelhardt Institute of Molecular Biology, Russian Academy of Sciences, Vavilov str. 32, 119991 Moscow, Russia; 3 Bach Institute of Biochemistry, Fundamentals of Biotechnology Federal Research Center, Russian Academy of Sciences, 119071 Moscow, Russia

**Keywords:** CRISPR/Cas system, genome editing, epigenome editing, schizophrenia, neurodevelopmental disorders

## Abstract

The study of diseases of the central nervous system (CNS) at the molecular level is challenging because of the complexity of neural circuits and the huge number of specialized cell types. Moreover, genomic association studies have revealed the complex genetic architecture of schizophrenia and other genetically determined mental disorders. Investigating such complex genetic architecture to decipher the molecular basis of CNS pathologies requires the use of high-throughput models such as cells and their derivatives. The time is coming for high-throughput genetic technologies based on CRISPR (Clustered Regularly Interspaced Short Palindromic Repeat)/Cas systems to manipulate multiple genomic targets. CRISPR/Cas systems provide the desired complexity, versatility, and flexibility to create novel genetic tools capable of both altering the DNA sequence and affecting its function at higher levels of genetic information flow. CRISPR/Cas tools make it possible to find and investigate the intricate relationship between the genotype and phenotype of neuronal cells. The purpose of this review is to discuss innovative CRISPR-based approaches for studying the molecular mechanisms of CNS pathologies using cellular models.

## 1. Introduction

Major psychiatric disorders, such as autism spectrum disorder (ASD), attention deficit hyperactivity disorder (ADHD), bipolar disorder (BD), depression, and schizophrenia (SZ), are characterized by complex etiopathogenetic mechanisms involving neuroanatomic abnormalities, biochemical imbalances, genetic and epigenetic changes [1]. Some the disorders, such as SZ, ASD, and BD, have a genetic component, with heritability typically estimated by twin studies to be 40% to 80%, with much of it due to common risk alleles [2]. Large-scale genetic studies have convincingly shown that distinct psychiatric disorders are likely to share common genetic risk variants [3]. It is important to note that their pathophysiology is multideterministic, as environmental factors interact with the polygenic architecture. In addition, even the physiological status of the patient influences the symptoms and signs of the diagnosed disorder [4]. Thus, the pathogenesis of psychiatric disorders can be considered a dynamic process with limited knowledge about spatial and temporal characteristics of the brain. For this reason, genomic data themselves have limited functional interpretations.

One way to assess the functionality of genetic risk variants is to identify and investigate their relationship with gene function and phenotypes at the cellular level. Thus, modeling NDDs in different cell types provides mechanistic insights into the connections between genetic risk variants and the pathogenesis of NDDs. Cellular models can also provide information about potential therapeutic strategies because they have the predictability to change the aberrant phenotype to a normal level by genetic intervention or drug administration. For example, predictive validity was confirmed for patient-derived induced pluripotent stem cells (iPSCs) differentiated in vitro into dentate gyrus-like hippocampal neurons. The phenotype of increased excitability of generated neurons in BD was selectively reversed by lithium treatment only in neurons derived from patients who also responded to lithium treatment [5].

Since iPSCs differentiate into neurons following the same trajectory as in the developing embryo, these cells are a convenient tool for studying neurodevelopmental disorders (NDD) such as SZ and ASD. Neurobiologists can compare iPSC-derived neurons from patients and control groups to try to identify the genetic and molecular basis underlying abnormal brain development and function. The observed neurodevelopmental changes may include an altered rate of cell proliferation and ability to migrate in neuronal progenitor cells (NPCs), abnormalities in neurite morphology, as well as disturbing expression dynamics of neuronal genes and pathways and electrophysiological properties of neurons. [6]. However, iPSCs obtained from different donors have large genetic and epigenetic differences, which also affects their ability to differentiate even when using the same protocol [7]. Isogenic iPSC-derived cell models can help overcome the limitations of intersubject cell models. Isogenic cells can be created from healthy donor cells by introducing potentially causative variants or from patient cells by curing pathogenic alleles. The original and mutated iPSC lines or their differentiated derivatives can then be compared to study the effects of the introduced mutations. Despite their low throughput, single-gene cellular models still have their advantages. They are useful for proving causality, performing mechanistic studies, and assessing the relative contribution of specific pathogenic variants or risk genes to the severity of NDD. For example, a 4 bp deletion in *DISC1* (Disrupted in Schizophrenia 1) was introduced into isogenic iPSCs, which then differentiated into forebrain neurons, and their synaptic dysfunction associated with synapse-related gene deregulation was studied [8]. Single-gene cell models are adequate for studying monogenic forms of mental disorders, such as Timothy syndrome, a monogenic form of ASD caused by loss-of-function mutations in *CACNA1C* (Calcium Voltage-Gated Channel Subunit Alpha1 C) [9]. In addition to iPSCs and their derivatives, neuropsychiatric studies have also used neuronal cell lines such as human neuroblastoma SH-SY5Y [10]. For instance, Unsicker et al. used SH-SY5Y to study the functional consequences of *SHANK2* mutations that have been identified in patients with ASD and mental retardation [11].

Genome editing technologies, generally the CRISPR/Cas systems, are used to produce isogenic cell lines and have contributed greatly to the functional analysis of prioritized risk variants associated with NDDs (e.g., [12]). The principle behind the CRISPR/Cas9 genome editing technology is as follows. The Cas9 endonuclease, after binding its single guide RNA (sgRNA), searches for genomic targets that are complementary to the sgRNA spacer and have a short NGG motif adjacent to the right side of the target sequence. When binding to the target, the spacer forms a heteroduplex with the complementary strand of the target, displacing the non-target DNA strand. Once the spacer:target annealing process is complete, the nuclease activity of the two Cas9 nickase domains are activated, resulting in a double-strand break (DSB) with blunt ends. This DSB can be repaired by the cell using the error-prone nonhomologous end-joining pathway or the homologous-directed repair pathway if a donor DNA fragment homologous to the edited site of the genome is provided [13].

The Cas9 endonuclease can be modified by inactivating one of its nickase domains and fusing with a deamination enzyme. Usually, cytosine or adenine deaminase produces a new type of genome editor called base editors (cytosine base editor, CBE, and adenine base editor, ABE, respectively) [14,15]. Cytosine is desaminated to deoxyuracil, which reads as thymine during DNA replication, and adenine is desaminated to inosine, which reads as guanine. Thus, CBE action converts C•G into a T•A base pair, and ABE action converts A•T to G•C base pair without toxic DSBs. Basic editors enable the creation and study of putative causal single nucleotide polymorphisms (SNPs) [16,17].

The complete inactivation of Cas9 nuclease domains (called dead Cas9 nucleases, dCas9) and its fusion with functional domains that act to modify DNA or histones results in epigenetic editors [18]. The fusion of dCas9 with transcription activation or repression domains creates artificial activators or repressors of transcription. Epigenetic editors change the expression of genes without changing their sequences and can be used to investigate genetic variants located in the *cis*- or *trans*- regulatory regions of NDDs risk genes.

CRISPR-based technologies have clear advantages over other genome editing technologies. In particular, they can be used to relatively rapidly create isogenic cell models with small changes, such as SNPs or deletions of large genomic regions. A major advantage of CRISPR/Cas systems is the possibility of simultaneous modification of multiple targets. This is important for the study of complex multigenic psychiatric disorders. In addition, CRISPR/Cas systems allow screening studies to rapidly identify multiple causal risk variants in a single experiment [19].

The purpose of this review is to present a selection of advances in genome and epigenome editing for studying the molecular basis of mental disorders.

## 2. Recent Insights into the Genetic Architecture of SZ and Other NDDs

Ever since early family studies confirmed that a genetic component contributes significantly to the development of mental disorders, it has been well-known that heritability plays an important role in the development of NDDs such as SZ and ASD. In addition to family, twin, and adoption studies, genome-wide association studies (GWAS) are a powerful hypothesis-free approach to searching for genetic NDDs risk loci. Risk loci mapped to genes, and genomic features can provide clues to unravel the pathogenic mechanism and provide the right choice of cell model and CRISPR/Cas tool for further validation and research.

In 2022, Trubetskoy et al. conducted the largest GWAS to date on SZ [20]. A collaborative effort between international consortia and the Psychiatric Genomics Consortium (PGC-3) analyzed case-control GWAS data that, in total, included the genomes of 306,011 individuals. The study identified 342 independent SNPs mapped to 287 distinct genomic loci that may increase the risk of SZ [20]. These SNPs are mostly noncoding and are associated with only a small increase in the risk of disease (the odds ratio is usually about 1.1). To find the possible causative variants, the authors prioritized variants and genes using a combination of fine mapping, transcriptomic analysis, and functional genomic annotations. As a result, three groups of genes were identified.

One group comprises the genes having at least one non-synonymous or untranslated region variant. This group includes genes such as *SLC39A8* (contains causative rs13107325), which regulates zinc and manganese uptake, interferon regulatory factor 3 (*IRF3*), transcription factor *KLF6*, the less functionally characterized *THAP8* (THAP Domain Containing 8) and *WSCD2* (WSC Domain Containing 2) and genes encoding E3 ubiquitin ligases, *PJA1* (Praja Ring Finger Ubiquitin Ligase 1) and *CUL9* (Cullin 9). Earlier studies also indicated the involvement of these genes in the pathogenesis of SZ and other NDDs. For example, *KLF6* is involved in the *NFATC2*-dependent gene regulatory network, the disruption of which leads to lipid abnormalities in *corpora callosa* in patients with SZ [21]. The *PJA1*-encoded ubiquitin ligase Ring-H2 is important for polyQ protein degradation [22] and thus has protective potential against neurodegenerative diseases and NDDs. Indeed, mutations in *PJA1* are associated with numerous X-linked NDDs, including neurodegenerative diseases [23] and ASD [24]. *WSCD2* has been found to be associated with extraversion [25] and temperament in ASD [26].

The second group comprises genes that are reliably explained by quantitative trait expression loci (eQTLs), i.e., variants that affect gene expression. Examples of genes with high scores associated with causal eQTLs are *ACE* (angiotensin-converting enzyme), *DCLK3* (Doublecortin Like Kinase 3), which is underexpressed in SZ and *SNAP91* (Synaptosome Associated Protein 91), which is overexpressed in SZ. Earlier studies also found an association between lower mRNA and ACE protein levels with an increased risk of SZ [27]. Although ACE is also present on neuronal membranes and is capable of cleaving several neuropeptides, suggesting its functions in the CNS [28], the molecular mechanism underlying the association of *ACE* and SZ remains unclear. Large-scale RNA-Seq analysis of high-quality postmortem brain samples from people with ASD, BD, SZ, and controls showed a significant association of changes in *DCLK3* and *SNAP91* expression with SZ and BD but not with ASD [29]. *SNAP91* encodes the clathrin envelope assembly protein AP180, which is involved in chemical synapse function [30]. Little is known about *DCLK3*, which encodes a serine/threonine protein kinase with a neuroprotective function [31].

The third group includes genes encoding proteins localized in synapses and functioning in them. Synaptic dysfunction is considered to be a central component of the pathophysiology of SZ [32]. Genes in this group encode voltage-gated calcium and chloride channels (*CACNA1C* and *CLCN3*), metabotropic receptors (glutamate (*GRM1*) and gamma-aminobutyric acid (*GABBR2*)) and the ligand-binding subunit of the N-methyl-d-aspartate (NMDA) receptor (*GRIN2A*). *SNAP91* also belongs to this group.

Heritability (h^2^) estimated from family and twin studies is 0.81, 0.80, and 0.75 for SZ, BD, and ASD, respectively [33]. However, SNP-based heritability (h^2^_SNP_) estimated by GWAS is 0.23, 0.25, and 0.17 for SZ, BD, and ASD, respectively [34]. The difference between h^2^ and h^2^_SNP_ can serve as an estimate of missing heritability. It has previously been suggested that missing heritability is a hidden heritability that cannot be evaluated because of the drawbacks of the genetic mapping approaches [33]. Thus, one way to identify the genetic components of missing heritability is to develop new approaches for obtaining and analyzing genetic data. For example, additional SZ and BD risk genes have been identified by collecting SNPs into large groups and accounting for their non-additive interaction effects [35]. This approach makes it possible to examine SNPs that have not reached genome-wide significance. Another way to identify components of missing heritability is to analyze rare genetic variants.

In order to identify rare genetic variants associated with SZ, the Schizophrenia Exome Sequencing Meta-Analysis (SCHEMA) consortium sequenced and analyzed the exomes of 24,248 people with SZ and 97,322 healthy controls, the largest data set to date on SZ exomes [36]. As a result, the authors identified 244 candidate genes carrying two types of disruptive ultra-rare coding variants. The first type includes truncated protein variants (PTVs), defined as variants with stop, frameshift, or essential splice donor or acceptor variants. The second type represents damaging missense variants. Of these, ten genes with the highest SZ risk were identified. They were annotated for the following functions: ion transport (*CACNA1G, GRIN2A,* and *GRIA3* (Glutamate Ionotropic Receptor AMPA Type Subunit 3)), neuronal migration and growth (*TRIO*), regulation of transcription (*SP4, RB1CC1,* and *SETD1A*), nuclear transport (*XPO7*) and ubiquitin ligation (*CUL1* and *HERC1*). Note that *CACNA1G, GRIN2A,* and *CUL1* are also identified in the GWAS mentioned above [20]. The overlap between GWAS and exome sequencing results supports the emerging consensus that rare and common genetic risk factors converge in the same molecular mechanisms of NDDs.

SZ risk genes could be shared with other NDDs. To explain the shared genetic background of phenotypically different NDDs, Singh et al. also examined differences in the mutations found in three risk genes *GRIN2A, CACNA1G,* and *TRIO*, which are associated with both the developmental delay/intellectual disability (DD/ID) and SZ [36]. It turned out that the PTVs of the selected genes were associated only with SZ, whereas the less damaging missense variants were associated with both SZ and DD/IDs. These data suggest that the degree to which genetic variants are deleterious for proteins responsible for neurodevelopment may influence the development of a particular disorder.

Molecular mechanisms associated with NDD risk genes identified in large-scale studies (e.g., *CUL9*, *SNAP91*, *SETD1A, CLCN3*) have been studied in cellular models using CRISPR/Cas9-based tools and are discussed in further sections of this review.

## 3. Applications of CRISPR-Based Genome Editing Technologies to Study SZ and Other NDDs

The identification of rare loss-of-function coding variants, such as PTVs, provides the most direct biologically interpretable links between gene function and the pathogenesis of a given mental illness. Loss-of-function gene mutations can be easily modeled by genetic disruption in cellular or animal models. Moreover, these mutations can be studied in both homozygous and heterozygous states. The latter is more relevant to human diseases because patients often have only one of their alleles disrupted. Genome editing with CRISPR-Cas systems facilitates the creation of isogenic models for subsequent molecular and phenotypic characterization to deepen our understanding of the mechanism of action of the mutation of interest.

The first part of genetic editing is the selection of the target gene. Many of the high-scored candidate SZ risk genes or other loci identified by PGC-3 are the subject of functional studies in various models created with CRISPR/Cas9 and other genome editing technologies. Selected studies examining the molecular mechanisms in which SZ risk genes are involved are listed in Table 1.

Cellular models are highly convenient for introducing desired genetic changes and studying the associated molecular mechanisms. Even non-neuronal cells, such as HEK293 and their derivatives, can be used to study the molecular mechanisms associated with mutations in genes associated with a high risk of SZ and other NDDs. HEK293, unlike neuronal cells, does not require specific culturing conditions and can be efficiently transfected. HEK293 has been used to study the pathological molecular mechanisms associated with *ARC* encoding activity-regulated cytoskeletal-associated protein (Arc/Arg3.1), a critical regulator of long-term synaptic plasticity involved in learning behavior [37]. *ARC*-KO HEK293 cells were obtained using the CRISPR/Cas9 system and examined at the transcriptomic and proteomic levels. The study revealed the deregulation of extracellular matrix (ECM)-related genes and proteins, with fibronectin (FN1) being the hub-building protein. The ECM disruption is in good agreement with the low adhesiveness of neurons derived from patients with SZ [51]. The study also revealed the deregulation of ICAM-2 (Intercellular Adhesion Molecule 2), a member of the ICAM family consisting of type I transmembrane glycoproteins. Interestingly, the deregulation of ICAM-1, another member of this protein family that functions as a leukocyte receptor, was found in a postmortem transcriptomic study of SZ patients [52]. Thus, these data link ECM disruption to neuroinflammation, which is a characteristic pathological process in SZ [53] and other NDDs [54]. Moreover, the genome of HEK293 and its derivatives can be easily edited because of the high transfection efficiency. Wang et al. could not obtain SH-SY5Y monoclonal cells with rs2270363 deletion because of low transfection efficiency. Therefore, the authors used HEK-293T to create a model cell line with rs2270363 deletion to study *NMRAL1* regulation [38]. However, not all SZ risk genes can be adequately studied in HEK293. For example, CRISPR/Cas9-mediated deletion of two downstream *FOXP2* enhancers in the SK-N-MC neuroblastoma cell line leads to impaired expression of *FOXP2* and its target genes, whereas deletion of the same enhancers in HEK293 has no effect [40]. This example shows that the data obtained in HEK293 lines need further verification in neuronal cell lines. Moreover, HEK293 lacks the constrictive validity characteristic for neuronal cell lines and therefore is not suitable for the electrophysiological, morphological, and other functional characterization of gene knockouts or their alleles related to the pathophysiology of the NDDs.

Neurons differentiated from CRISPR/Cas9-edited human embryonic stem cells (hESCs) represent a more relevant cellular model for studying pathogenic molecular mechanisms associated with SZ. So, Sanders and colleagues obtained cortical excitatory neurons from hESCs with homozygous loss-of-function mutations in the *DLG2* (Discs Large MAGUK Scaffold Protein 2) gene using the CRISPR-Cas9 system [41]. *DLG2* mutations are found in patients with SZ and ASD and likely cause dysfunction of developmental signaling pathways relevant to NDDs pathophysiology. *DLG2* mutations are found in patients with ASD and ALS and likely cause dysfunction of developmental signaling pathways relevant to the pathophysiology of NDD. *DLG2* disruption leads to significant changes in the transcriptome, reaching about 60% of all expressed genes at some time points. Mutant hESCs show a significant delay in differentiation, being only 15% of the wild-type level by day 30, as assessed by the detection of cortical neuronal markers. Moreover, a number of genes related to neuronal morphology, migration, and action potential generation are downregulated in mutant cells [41]. Interestingly, SZ risk genes are enriched among the deregulated genes. These data indicate that *DLG2*, as a regulator of neurodevelopmental transcriptional programs, is a high-risk gene for NDDs that links to many other risk genes.

*CUL9* is another gene identified as an SZ risk gene [20], but knockout studies have failed to identify its function in neurodevelopment. CUL9 is a poorly studied member of the largest family of E3 ubiquitin ligases. Known as Cullin RING ligases, which are part of the ubiquitin-proteasome protein degradation system [55]. CUL9 is highly expressed in the brain, especially in the cerebral cortex, indicating its important role in CNS function. However, only a few CUL9 substrates are known, which limits the understanding of its role. Human iPSCs (hiPSCs) with CRISPR/Cas9-mediated *CUL9* knockout exhibit pluripotent properties and the ability to differentiate into NPCs, then into neuronal progenitor cells, and then into cortical neurons as wild-type hiPSCs [42]. The only observed defect in vitro neurodevelopment of *CUL9*-KO hiPSCs is abnormal neuronal rosette formation. Differential proteomic analysis revealed the upregulation of metabolic enzymes in *CUL9*-KO iPSCs and NPCs. However, subsequent experiments did not confirm changes in relevant metabolic processes. Neuronal transcription factors CUX1 and SOX3 were significantly upregulated in *CUL9*-KO NPCs at the mRNA level, but no significant changes were found at the protein level. These data are consistent with *CUL9*-KO mice being viable and showing no significant signs of abnormal neurodevelopment [56]. Taken together, these data suggest that the loss of CUL9 function can be easily compensated by other E3 ligases. Nonetheless, a recent family study combined with whole exome sequencing has identified a variant (43181034 T > G) in the splicing region on exon 27 of *CUL9* that is associated with multiple sclerosis, a neurological autoimmune disease [57]. It is possible that CUL9 still plays an important role in neurodevelopment and/or neuroinflammation. Given the accumulation of CUL9 during neurodevelopment [42], studies on its overexpression or investigating its mutant variants instead of knockout may help reveal its functions.

*STAG1* and *STAG2* (Stromal Antigen 1 and 2) encode subunits of the cohesion complex important for 3D genome organization, gene expression, and embryonic development [58]. Exome analysis of SZ patients revealed rare variants in *STAG1* [36]. Moreover, mutations in *STAG1* [59] or *STAG2* [60] are significantly associated with developmental delay/intellectual disability (DD/ID). To elucidate the possible connection of STAG1 and STAG2 malfunction with neurodevelopment, the mouse embryonic stem cells (mESCs) *Stag1^−/−^* and *Stag2^−/−^* were generated using CRISPR/Cas9 tools [47]. The cells tolerate the loss of either of these genes well. It turned out that Stag1 and Stag2 are localized in the same locations throughout the genome but are never observed in the same complex. The absence of either Stag protein does not affect the distribution of cohesin in the genome. Transcriptome analysis of *Stag1^−/−^* and *Stag2^−/−^* mESCs compared to isogenic mESCs revealed deregulation of a large number of genes that are classified in the following GO terms related to neurodevelopment: nervous system development, neurogenesis, neuron development, neuron differentiation, neuron projection development, neuron projection morphogenesis, regulation of neurogenesis, regulation of neuron differentiation. These data confirm the association of Stag1 and Stag2 with neurodevelopmental disorders. However, even heterozygous deletion of any of these genes is lethal in embryogenesis. Therefore, models carrying pathogenic alleles rather than knockouts should be used to further study neurodevelopmental abnormalities associated with *STAG* genes.

In addition to the protein-coding genes, there are a number of common SZ risk variants identified in protein-noncoding loci that are being studied in neuronal cell lines. For example, the SNP (rs2270363: G>A) at 16p13.3 determines the risk of SZ by deregulating *NMRAL1*. *NMRAL1* encodes a NmrA-like transcription factor that regulates cellular metabolism by sensing intracellular redox balance through NADPH binding [61]. rs2270363 is located in the E-box element of the *NMRAL1* promoter and disrupts the binding of the three bHLHZ transcription factors, USF1, MAX, and MXI1. Wang and colleagues used CRISPR-Cas9-mediated editing in HEK-293T to confirm that the rs2270363-containing locus affects *NMRAL1* expression [38]. Moreover, they found it decreased *NMRAL1* expression in the postmortem brains of patients with SZ carrying rs2270363. Using human SH-SY5Y and SK-N-SH cell lines, mouse neural stem cells (NSCs), and rat primary cortical neurons, the authors showed that deregulation of *NMRAL1* expression affects the proliferation and differentiation of NSCs and significantly reduces the density of dendritic spines on neurons. Thus, rs2270363 is responsible for the risk of SZ development by impairing neurodevelopment and morphogenesis of dendritic spines, two characteristic features of SZ pathophysiology.

Another example is the regulatory variants rs796364 and rs281759 at 2q33.1, which are associated with the risk of SZ by modulating the expression of the distal *TYW5* (TRNA-YW Synthesizing Protein 5) gene [39]. This gene encodes a major tRNA hydroxylase involved in brain epigenetic modification [62]. SNPs are within the enhancer, which physically interacts with the *TYW5* gene and disrupts *CTCF* (CCCTC-Binding Factor), *RAD21* (Radiation Sensitivity 21), and *FOXP2* (Forkhead Box P2) binding. The knockout of rs796364 and rs281759 in HEK293T, SH-SY5Y, and SK-N-SH cell lines using the CRISPR/Cas9 double-sgRNA system confirmed the regulatory role of these SNPs on *TYW5* expression. Modeling the upregulation of *TYW5* in SZ, the authors overexpressed *TYW5* in mouse NSCs and primary rat neurons and found significant changes in NSCs proliferation and differentiation as well as dendritic spine density in neurons. Moreover, transcriptome analysis showed that *TYW5* deregulation affects schizophrenia-related pathways. A recent comprehensive and integrative analysis confirmed that *TYW5* is a risk gene for SZ [63]. These data strongly suggest that *TYW5* deregulation is a risk factor for SZ.

The use of patient-derived iPSCs offers great opportunities for the study of human neurodegenerative and psychiatric diseases [64]. iPSCs were initially used to model diseases with highly pervasive genetic variants with a large phenotypic effect. By now, their application has expanded to the field of modeling psychiatric diseases and generating patient-specific organoids. The ability of iPSC-derived neurons to reproduce fundamental neuronal functions, including conducting action potentials and releasing neurotransmitters, has led to the development of functional analysis of variants associated with SZ. A recent example is a study of *SETD1A* in iPSC-derived glutamatergic/GABAergic neurons [46]. The authors showed that *SETD1A* haploinsufficiency leads to transcriptomic changes that are most significantly associated with SZ and BD and phenotypically manifested as increased dendrite complexity and a functional increase in neuronal network burst activity. A more detailed analysis of the deregulated genes revealed that *SETD1A* haploinsufficiency leads to hyperactivation of the cAMP/PKA/CREB pathway. These results were confirmed by experiments with the PKA (Protein Kinase A) inhibitors H89 and KT5720, which normalize *SETD1A*^+/−^ network activity.

Cellular models and neural networks helped to reveal many important molecular and cellular mechanisms of NDD. However, they cannot be used to study higher levels of organization characteristic of brain structure as well as its development. This necessitates the creation of another type of model that would mimic the brain features and development. Cerebral organoids derived from hiPSCs have become such a model. They have an advanced three-dimensional structure (forebrain, midbrain, and hindbrain) and a complex organization similar to the human fetal brain [65].

Fragile X syndrome (FXS) is an X-linked NDD that, in some cases, meets the diagnostic criteria for autism. FXS is caused by transcriptional silencing of the *FMR1* (Fragile X Mental Retardation 1) gene due to the expansion of >200 CGG repeats in the *FMR1* promoter region [66]. In order to meet the need for an adequate model to study and treat FXS, cortical organoids were grown from CRISPR/Cas9-mediated *FMR1* knockout hiPSCs [49]. *FMR1* KO organoids show increased size and number of astrocytes. These data are consistent with impaired human NPC differentiation [67] and altered cortical cytoarchitecture in the *FMR1* KO mouse model [68] and allow the authors to suggest that *FMR1* is responsible for the proper balance of neural and glial components during cortex development. In another study, FXS forebrain organoids were derived from patient-derived iPSCs [66]. Such organoids showed a wider range of defects, such as the decreased proliferation of NPCs, dysregulation of neuronal differentiation, increased synapse formation, enhanced neuronal excitability, and reduced production of GABAergic neurons. In contrast to the study [66], organoid size was the same as in controls, and although the radial glia layer was increased, no increase in astrocytes was observed. The differences between the *FMR1* KO organoids and those from FXS patients indicate that the consequences of CRISPR-mediated inactivation of *FMR1* should be investigated with caution. Interestingly, FMR1 direct targets are enriched in SZ- and ASD-related genes [69]. In particular, the FMR1 target *CHD2* (encoding Chromodomain Helicase DNA-binding protein 2) has a critical function in neurogenesis and, in particular, in the generation of GABAergic interneurons [70] and is associated with ASD [71]. These findings explain why some patients with FXS may exhibit symptoms of ASD.

In another study, mutant cerebral organoids [50] were grown from iPSCs obtained from a patient with a de novo missense mutation in the *AUTS2* gene (c.1600 A>C, T534P). Mutant organoids show significant growth retardation, which correlates well with the patient′s microcephaly. Growth retardation is associated with a proliferative deficit in NPCs. Single-cell transcriptome analysis showed that the *AUTS2* mutation was associated with dysregulation of WNT-β-catenin signaling, chromatin modification, and gliogenesis. The authors also grew cerebral organoids from the same iPSCs but with CRISPR/Cas9-corrected mutation. *AUTS2* correction restored NPCs proliferative activity and organoid growth, indicating the therapeutic potential of the CRISPR/Cas9 system in correcting NDDs.

The effect of rs4702 on *FURIN* expression was evaluated in 3D cortical spheroids (hCSs) and subpallial spheroids (hSSs) derived from CRISPR/Cas9-edited hiPSCs [12]. However, the authors found slightly reduced (in the case of hSSs) or unchanged (in the case of hCSs) expression of *FURIN* compared to neurons. Such results illustrate a limitation of organoids: due to cell heterogeneity, they can conceal the cell-specific expression pattern of the genes of interest. This limitation can be overcome by single-cell transcriptome analysis, as was performed in the study of the *AUTS2* mutation [50].

There are other difficulties in using organoids to model brain development, structure, and function. Among them are incomplete cellular composition and incomplete anatomy, including the absence of blood vessels important for brain development. To overcome this limitation, researchers are improving methods for growing brain organoids with blood vessels [72,73]. These vascularized brain organoids showed the presence of a functionally active blood-brain barrier and microglia responding to immune stimulation. Vascularized brain organoids can be used to study neurovascular diseases and injuries, as well as for drug screening and brain-vessels pharmacodynamics.

Assembloids represent the next level of 3D brain models with increased cellular composition and structural complexity [74]. A recently developed protocol for creating cortico-striatal assembloids [75] should help investigate corticostriatal connections that are affected in neuropsychiatric diseases, including ASD [76] and SZ [77].

The studies discussed above investigate individual genes. However, SZ and other NDDs are complex multigenic disorders, so studying individual targets limits the ability to identify causative variants and decreases the depth of our understanding of the complex mechanisms characteristic of NDDs. The CRISPR knockout (KO) screening technology allows multiple gene targeting in a single experiment and greatly expands the possibilities of identifying causal variants and investigating multigenic molecular mechanisms. Since neurons are non-dividing and hard to transfect cells, the CRISPR KO screenings are applied to iPSC-derived cellular models. Moreover, Cas9 doxycycline controllable systems are used [78,79] to overcome Cas9 toxicity to iPSCs [80]. CRISPR KO screenings are usually performed for a functionally related group of genes, for example, kinases [78] or high-risk NDDs genes [79], to exclude genes irrelevant to the study, decrease the number of false-positive hits, decrease the loss of edited cells with lowered competitive fitness and thereby increase the sensitivity of the analysis. So, CRISPR KO screening of 425 genes associated with the risk of ASD and other NDDs was performed in human forebrain assembloids (hFAs) to search for genes involved in the development and migration of cortical interneurons [79]. hFAs were derived from hiPSCs derived from human subpallial organoids (hSO) and human cortical organoids. As a result, loss of *SMAD4* (SMAD Family Member 2) or *CSDE1* (Cold Shock Domain Containing E1) disturbs subpallium differentiation and decreases hSO size. Loss of *TERF2* (Telomeric Repeat Binding Factor 2) and *LNPK* (Lunapark, ER Junction Formation Factor) impairs interneuron migration but does not affect subpallium differentiation. The study also showed that not all high-ranked hits of the primary screening could then be validated. Thus, it should be noted that CRISPR KO screening identifies candidate genes that need further validation in single-gene models.

GWAS or exome sequencing studies mostly find small structural variants, such as single base changes in candidate genes or other genomic loci. Obviously, a candidate gene knockout is a very harsh intervention and does not allow the genetic variant itself to be examined. Moreover, knockouts cannot be used to study essential genes. To get closer to the true depth and complexity of NDDs, we need more delicate and precise tools to create models with changes at a single base level. CRISPR/Cas technology offers such tools called base editors. Cytosine base editing screens are used, for example, in cancer research to generate or validate ClinVar pathogenic variants to further study their molecular mechanisms of action [16,17]. Although such screenings have not yet been performed in the case of NDDs, they will certainly help to find and validate causal variants of NDDs to better understand the genetic architecture of NDDs using iPSC-derived neuronal models.

## 4. Epigenetics of SZ and Other NDDs

Some authors believe that the genetic component cannot explain the entire heritability of SZ and other NDDs. Since all psychiatric concordance rates are well below 100% for monozygotic twins [81], it has been suggested that another important component of missed heritability is epigenetic inheritance [82]. Recent advances in functional genomics show that genetic variations and epigenetic dysregulation of transcriptional networks are associated with neuropsychiatric disorders [83]. Therefore, we discuss recent data concerning the abnormal epigenetic mechanisms involved in the pathogenesis of SZ and other NDDs.

A set of transcriptional programs controls the selective expression of neuronal identity genes during brain development. Gene expression programs are coordinated in part by basic epigenetic mechanisms such as DNA methylation/hydroxymethylation, posttranslational modifications of histone proteins, nucleosome remodeling/re-positioning, and regulation of non-coding RNAs [84]. To uncover the role of epigenetic factors in psychiatric disorders, researchers conduct epigenome-wide association studies (EWAS) [85]. It has been shown previously that genetic variants can affect the level of DNA methylation at genomic CpG-rich loci. These genetic variants are called quantitative methylation trait loci (mQTLs) [86]. Most EWAS studies examine the relationship between DNA methylation and NDDs and use whole-genome bisulfite sequencing (WGBS) to map methylated cytosines with single-base resolution [87]. The disadvantages of bisulfite sequencing are well known, so WGBS is constantly being improved. Using WGBS, Mandell et al. conducted an extensive search for mQTLs among SNPs associated with the risk of SZ in postmortem brains [88]. As a result, they found that the proportion of mQTLs among the selected SNPs could be as high as 93%. This indicates that the contribution of mQTLs among the variants associated with SZ is much broader than traditionally thought. In another study, using a Summary data-based Mendelian Randomization (SMR) method, the authors integrated mQTL and GWAS to identify three new promising candidates for high risk of SZ rs55742290-cg00376283-*ABCB9* (ATP Binding Cassette Subfamily B Member 9), rs3765971-cg00546117-*RERE* (Arginine-Glutamic Acid Dipeptide Repeats) and rs7293091-cg21663219-*TNFRSF13C* (TNF Receptor Superfamily Member 13C) [89]. Notably, the integrated SMR method identified rs7293091 as associated with SZ, although this variant was not found by GWAS, which suggests the applicability of the SMR method for revealing missing heritability. By combining data from several other GWAS and Haploreg v4 databases, the authors proposed the following molecular mechanism for rs55742290 located on the *ARL6IP4* (ADP Ribosylation Factor Like GTPase 6 Interacting Protein 4) promoter for further validation in functional studies. The [C] rs55742290 risk allele decreases methylation at the CpG site of cg00376283, thereby disrupting repressor(s) binding and increasing *C12orf65* gene expression, which affects cognitive performance and increases the risk of developing SZ [89]. The proposed mechanism can be further validated in isogenic cell models edited by CRISPR/Cas9.

Altered serotonergic gene expression caused by genetic or epigenetic factors has been observed in neuropsychiatric disorders, including depression, stress-related anxiety disorders, obsessive-compulsive disorder, ASD, and SZ [90]. Interestingly, chromatin accessibility regulators of postmitotic neurons determine the molecular and morphological features of specialized neurons. Zhang et al. showed that the regulatory factors Pet1 and Lmx1b play a key role in the formation of mature serotonin (5-HT) neurons from the Pet1 subtype. They directly control the availability of 5-HT-specific cis-regulatory elements associated with genes encoding terminal effectors of 5-HT identity and neurotransmission. The paper states that unique distal enhancers define the Pet1 neuronal lineage, which produces 5-HT neurons in mice [91]. Mature neurons show enriched gene expression with variants associated with autism and other NDDs. Because autism-related genes are often involved in chromatin remodeling and transcriptional regulation, their dysfunction during development may contribute to disease pathogenesis [92]. The critical role of chromatin remodeling and histone modification mechanisms is also observed in neurons both during development and in adulthood in response to external stimuli. Through epigenetic regulation of neuronal gene expression, environmental stimuli are transferred to neurobiological substrates capable of controlling behavior both in health and disease. Disease-related changes in the local chromatin structure at specific gene promoters can induce transcriptional changes that are directly related to the underlying etiology or secondary events in the pathophysiology of the disease. This is related to the transcriptional memory of neurons. Importantly, in postmitotic cells, transcriptomes remain dynamic to drive structural and functional changes as mature neurons integrate multiple and diverse signals to support different forms of plasticity [93].

In the adult brain, specific gene expression programs are altered by neuronal activity and behavioral experience, and these changes are crucial for adaptive behavior [94]. Dysregulation of gene expression programs both during development and in the adult brain is associated with numerous neuropsychiatric diseases such as addiction [95], depression [96], and SZ [97]. In recent years, epigenetic studies in the postmortem brain in SZ have mainly focused on identifying differentially methylated sites and genes in the cortex and other brain regions [98]. The results of these studies suggest that hypermethylation of the promoter of *DUSP22* encoding double specificity phosphatase 22 [98] and differential methylation in the *MAD1L1* (Mitotic Arrest Deficient 1-like 1) coding region [99] are SZ risk factors. Moreover, the *MAD1L1* differential methylation sites colocalize with the transcript quantitative trait loci and the GWAS signal, which strongly suggests the contribution of epigenetic dysregulation of *MAD1L1* to the pathogenesis of SZ. The reviewed examples of epigenetic dysregulation of risk genes for SZ and other NDDs show that epigenetics is probably the missing link between genetic variations and physiological changes in the CNS of patients with NDDs.

## 5. Application of CRISPR-Based Epigenetic Editors to Study SZ and Other NDDs

The development of nuclease-free Cas9 derivatives opens up a series of CRISPR/Cas tools aimed at manipulating epigenetics, i.e., DNA and histone modifications, and creating artificial transcription factors. CRISPR/dCas9 epigenetic editors allow manipulation of neuron-specific transcriptional programs to identify epigenetic hallmarks of NDDs and link them to genetic risk loci (Table 2). Currently, the development of more efficient epigenetic CRISPR/Cas editors, for example, CRISPR/Cas9 repressors, is ongoing. The most commonly used dCas9-KRAB repressor contains the KRAB domain from KOX1 (ZNF10, Zinc Finger Protein 10) [100]. However, the human genome encodes more than 350 KRAB-domain-containing proteins [101]. Recently, Nader Alerasul and colleagues tested the repressor activity of 57 KRAB domains and identified the KRAB domain ZIM3 as an extremely potent repressor [102]. They showed that ZIM3 KRAB-dCas9 is superior to existing KOX1-based KRAB repressors. The activity of the dCas9-KRAB or dCas9-ZIM3 systems can be further enhanced by adding MeCP2 (Methyl CpG-Binding Protein 2), which binds to methylated DNA and recruits corepressor Sin3a and histone deacetylases (HDACs) [103,104]. De novo DNA methyltransferases Dnmt3a and Dnmt3L fused to dCas9 can be used to establish long-term and long-range methylation of DNA loci [105]. Another study showed that some loci could be silenced by the histone methyltransferase EZH2 but not by the KRAB methyltransferase fused to dCas9 [106]. The highest levels of epigenetic silencing of target genes can be achieved when histone and DNA methyltransferase activities are combined as separate chimeric proteins, such as dCas9-Dnmt3a-Dnmt3L + dCas9-Ezh2 or dCas9-Dnmt3a-Dnmt3L + dCas9-KRAB) [107] or as a single KRAB-dCas9-Dnmt3a-Dnmt3L fusion protein [108].

Artificial CRISPR/Cas9 transcription factors are created by fusing transactivation domains to dCas9. Examples of transactivation domains used are the transactivating subunit of nuclear factor-κB (p65), the VP16 activation domain of herpes simplex virus (VP16), and four repeats of the VP16 activation domain (VP64). The strength of CRISPR activators can be increased by using a combination of transactivation domains such as VPR (consisting of VP64, p65, and RTa) or arrays of activation domains in the SunTag system (VP64 array recruited to dCas9) [119]. Other examples of epigenetic CRISPR editors are fusions of dCas9 with the catalytic domains of methylcytosine dioxygenase TET1 or human histone acetyltransferases p300 or LSD-1 [120].

The applications of epigenetic editors to understand the epigenetic mechanisms involved in SZ and other NDDs can be divided into two directions of research. The first direction is the application of CRISPR/Cas9-based tools to manipulate the activity of genome-encoded DNA methyltransferases and other natural epigenetic mechanisms to elucidate their role in the regulation of risk genes for SZ and other NDDs. The second direction is the use of artificial epigenetic editors based on CRISPR/Cas system to directly influence the expression of target genes.

**Figure 1:**
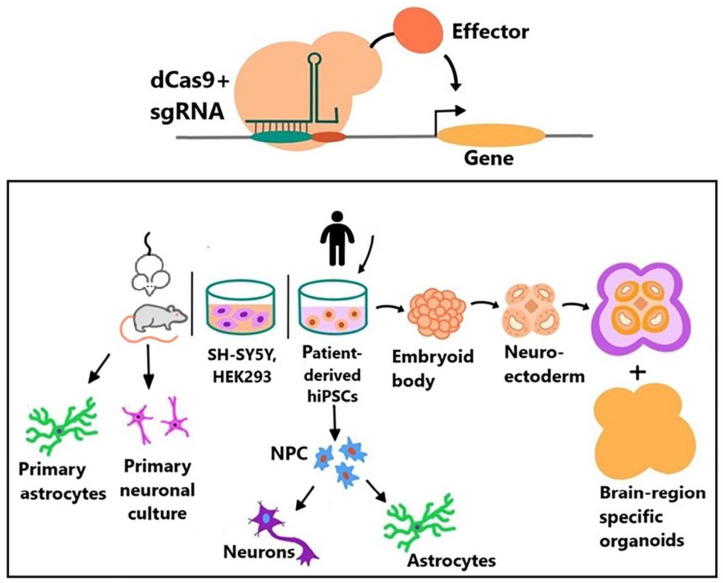
Models were used to study epigenetics of neurodevelopmental disorders.

Next, we provide recent examples of the use of CRISPR-based epigenetic editors to study epigenetic mechanisms associated with NDD. The relevant models used for epigenetic studies are summarized in Figure 1. The dCas9-KRAB-mediated repression of *PTEN* (Phosphatase And Tensin Homolog) transcription in the rat PC-12 cell line, HEK-293T, and iPSC-derived neurons was used to develop an effective approach to CNS regeneration after damage [109]. The dCas9-KRAB repressor recruited near the *PTEN* promoter causes methylation and deacetylation of histone H3 at the *PTEN* promoter, followed by strong and specific inhibition of *PTEN* transcription. PC-12 cells with an NGF-stimulated neuronal phenotype exhibit an average increase in neurite length when dCas9-KRAB-mediated *PTEN* repression occurs. This system showed better results compared to the previously used shRNA-mediated repression system.

An optimized CRISPR-based dual lentiviral expression system encoding the dCas9-KRAB-MeCP2 repressor showed effective repression of the *BDNF* (Brain-Derived Neurotrophic Factor) gene in primary cultures of rat hippocampal neurons [111]. Expression of dCas9-KRAB-MeCP2 was driven by the human neuron-selective *SYN1* promoter. The dCas9-KRAB-MeCP2 repressor demonstrated transcript-selective knockdown and outperformed commonly used RNAi knockdown methods in neuronal systems.

Optimized systems of tightly regulated Cre-dependent CRISPRa and CRISPRi were developed [110]. The intron-containing Cre-dependent activator SVI-dIO-dCas9-VPR and the repressor SVI-dIO-dCas9-KRAB-MeCP2 showed finer regulation and less leaky induction compared with classical Cre-dependent transcriptional CRISPR regulators in HEK293T models and primary animal neuronal cultures. The ability of these systems for multiple gene activation was demonstrated for the *GRM2* (Glutamate Metabotropic Receptor 2), *Tent5b, Fos, Sstr2,* and *Gadd45b* genes. These studies illustrate the advantage of the CRISPR/Cas9 system in working with multiple targets.

Genes related to neurotransmitter secretion *Syt1* (Synaptotagmin I), *Vamp2* (Vesicle Associated Membrane Protein 2), *Stx1a* (Syntaxin 1A), and *Snap25* (Synaptosome Associated Protein 25) were repressed in cultured mouse hippocampal neurons using dCas9-KRAB with the efficiency of over 90% [117]. Whole-cell patch-clamp recording analysis showed that repression of any of these genes leads to a significant decrease in the amplitude of excitatory postsynaptic currents (EPSCs). Using the neuron-specific promoters pCaMKIIα and pVGAT, dCas9-KRAB together with sgRNA against *Syt1* were selectively expressed in glutamatergic or GABAergic neurons to control their activity in vivo and assess the learning ability of animals. Moreover, the authors showed that the CRISPR repressor can highly efficiently knockdown five genes simultaneously *Syt1*, *Vamp2*, *Snap25*, *Stx1a,* and *Stx1b* in vivo, revealing the potential of the CRISPRi system to work with multiple targets [117]. In addition, these experiments demonstrated the possibility of the successful application of CRISPR repressor systems in the brain.

In another study, Dravet syndrome was corrected by dCas9-mediated activation of the *SCN1A* (Sodium Voltage-Gated Channel Alpha Subunit 1) gene in primary mouse hippocampal and cortical neurons. [112]. Mouse cells cotransduced with lentiviral constructs expressing the activator dCas9-VP160 and optimal sgRNA against the *SCN1A* proximal promoter (*Scn1a*-dCas9A system) showed increased *SCN1A* expression, increased Nav1.1 channel level and enhanced excitability. The results obtained in cellular models were successfully reproduced in *Scn1a^+/−^* mutant mice. Adeno-associated viral (AAV) vectors were used to efficiently deliver the CRISPR activator into brain cells. In order to fit within AAV packaging limits, a CRISPR activator with a smaller VP16 activation domain was used. Mice transduced with Scn1a-dCas9A showed a significant reduction in defects in parvalbumin interneurons and an increase in the threshold temperature for hyperthermia-induced seizures compared to mice transduced with the control Ctrl-dCas9A system [112]. CRISPR-based epigenetic editors offer an alternative approach to restore *FMR1* expression in patients with FXS. Liu et al. used dCas9 fused to the catalytic domain of Tet1 (Tet Methylcytosine Dioxygenase 1) to demethylate CGG repeats at the *FMR1* locus in iPSCs obtained from patients with FXS [113]. Demethylation (CGG)n restores *FMR1* expression (about 90% of the wild-type level) by changing the chromatin state from repressed to activated. In neurons differentiated from epigenetically edited FXS iPSCs, the firing rate decreased to wild-type levels. Importantly, the demethylation state (CGG)n in epigenetically edited neurons was maintained for at least two weeks in vitro and 3 months in vivo after transplantation into mouse brains. A 45% level of *FMR1* reactivation was achieved in postmitotic FXS neurons by direct transduction of lentiviral constructs expressing dCas9-Tet1 and dC-T/CGG sgRNA, and this was sufficient to reverse the spontaneous hyperactivity of FXS neurons. These authors also obtained FXS neurons with (CGG)n deleted using the CRISPR/Cas9 system and found that the genetically edited neurons exhibited almost the same restored phenotype as the epigenetically edited neurons [113]. These data suggest that epigenetic editing is as powerful as genome editing. However, compared to genome editing, epigenetic editing is less labor- and time-intensive and has reduced off-target activity, which is inherent for Cas9-based genome editors [121].

The helix-loop-helix transcription factor TCF4 is important for committing neuronal lineages and neuronal function during brain development. SNPs and somatic mutations in the *TCF4* gene are associated with SZ, BD, and ASD, Pitt-Hopkins syndrome (PTHS) [122]. In order to study brain abnormalities upon *TCF4* dysfunction, cortical organoids from a PTHS patient with a causative heterozygous *TCF4* mutation were obtained [118]. PTHS organoids show reduced expression of *TCF4* (at the mRNA and protein level), the NPC marker SOX2, and the neuronal marker MAP2. Morphologically, PTHS organoids are smaller in size, have fewer rosette-like cell aggregates, some have a polarized structure, and the density of neural precursors is significantly reduced compared to control organoids. Abnormalities in PTHS organoids were confirmed in a postmortem sample of the PTHS cortex. PTHS organoids cotransduced by three lentiviral constructs encoding the three-component CRISPRa system [123], which increases *TCF4* expression, showed corrected expression levels of *CDKN2A, MAP2, GADD45G,* and *SOX3*. Importantly, PTHS phenotypic abnormalities were corrected in these organoids, normal rosette-forming spheroids devoid of aberrant polarization were restored, and the number of immature neurons was reduced to normal levels. These data demonstrate the potential of the CRISPRa system in reversing *TCF4*-associated neurodevelopmental pathology.

CRISPR-based interference/activation screenings (CRISPR i/a) can be a valuable tool in epigenetic studies of polygenic NDDs. Like CRISPR KO screening, they allow the search for causative variants and risk genes within a single experiment. Since the CRISPR i/a systems do not damage DNA and do not affect neuronal differentiation and activity nonspecifically [124], they are an adequate alternative for CRISPR KO screenings. In an elegant study, the authors used the tetracycline-inducible *NGN2* (Neurogenin 2) gene for controlled differentiation of iPSCs into neurons [124]. The *NGN2*-inducible system helps to find out that genes related to sterol metabolism (e.g., *HMGCR* encoding HMG-CoA Reductase) are important for the survival of neurons but not iPSCs. Interestingly, this system also helps to identify genes whose knockdown increases neuronal survival, such as *MAP3K12* (Mitogen-Activated Protein Kinase Kinase Kinase 12), *MAPK8* (Mitogen-Activated Protein Kinase 8), *CDKN1C* (Cyclin Dependent Kinase Inhibitor 1C) and *EIF2AK3* (Eukaryotic Translation Initiation Factor 2 Alpha Kinase 3). Moreover, screening performed in co-cultures of neurons and astrocytes revealed two groups of genes. The first group is the metabolic genes *PPCDC* (Phosphopantothenoylcysteine Decarboxylase), *UROD* (Uroporphyrinogen Decarboxylase), and *MAT2A* (Methionine Adenosyltransferase 2A), whose knockdown is less toxic in the presence of astrocytes. The second group consists of the metabolic and regulatory genes *MMAB* (Metabolism Of Cobalamin Associated B), *UBA1* (Ubiquitin Like Modifier Activating Enzyme 1), and *PPP2R2A* (Protein Phosphatase 2 Regulatory Subunit B alpha), whose knockdown is more toxic to neurons in the presence of astrocytes. [124]. These data indicate the importance of interactions between different cell types in the brain, which may be missed by screening with single cell types. Thus, CRISPR screening in co-cultures of neurons with glial cells is necessary to study NDDs because glia contributes significantly to synaptic homeostasis and neuroinflammation. [125].

Neuroinflammation is an important component of the complex pathology of SZ and other NDDs [126]. Proinflammatory cytokines, such as a combination of IL-1α, TNF, and C1q, stimulate astrocytes to exhibit neurotoxic activity [127]. CRISPRi screening was applied to hiPSC-derived astrocytes to identify cellular pathways responding to IL-1α+TNF+C1q [128]. Based on computational master regulator analysis, authors defined candidate genes encoding transcription factors, kinases, or phosphatases and performed pooled CRISPRi screening with a custom sgRNA library. This approach has led to the identification of gene sets representing known inflammatory pathways (e.g., the NF-κB pathway) as well as pathways with no obvious connection to the pro-inflammatory activity of astrocytes (mTOR pathway (*MTOR*, *LAMTOR3*, *LATS2,* and *FOXK1*), the glucocorticoid receptor pathway (*NR3C1*), the actin cytoskeleton (*ARPC3* and *ACTR2*)). To gain a deeper understanding of the genes whose expression was altered by the knockdown of the corresponding regulators, the authors performed CRISPR drop sequencing, which combines CRISPRi perturbations with single-cell transcriptomics [129]. After subsequent confirmatory experiments, a hierarchical response to proinflammatory cytokines was constructed that resulted in two different inflammatory-reactive states of astrocytes.

CRISPRi screens assess genes and pathways for their disruption. In contrast, some genetic variants lead to the upregulation of genes and pathways. CRISPRa screens are more suitable for studying the functional significance of such variants. CRISPRa screening was applied to NGN2-induced glutamatergic neurons to investigate the molecular mechanisms associated with the overexpression of twelve upregulated high-risk genes of SZ (*CALN1, CLCN3, FES, INO80E, NAGA, NEK4, PLCL1, SF3B1, TMEM219, UBE2Q2L, ZNF823,* and *ZNF804A*) [130]. In order to track the developmental pathways affected by the overexpression of these genes; transcriptomic studies were performed at two different time points. As a result, the authors showed that the common effects converge on developmental pathways involved in patterning, regionalization and growth, neuroactive ligand-receptor signaling, and voltage-gated ion channel activity. Moreover, in silico modeling studies have shown that convergence increases with increasing polygenicity, confirming the polygenic additive model of SZ.

## 6. Conclusions and Perspectives

The collective efforts of international consortia and laboratory teams have made it possible to identify a large number of priority genes and variants responsible for the significant risk of the development of SZ and other NDDs. The identification of causative variants can be performed using various cellular models obtained by applying CRISPR-based genetic and epigenetic editors. CRISPR screening technology combined with transcriptomic studies can reveal pathways associated with disrupted high-risk NDDs genes leading to characteristic disease phenotypes. We believe that the combined use of CRISPR/Cas strategies to manipulate genome architecture, genome sequence, and epigenome has great potential to decipher complex gene regulatory networks in neuronal circuits and discover links between the complex genetic architecture of mental disorders and their phenotypes. To deepen the mechanistic understanding of the network dysregulation underlying the major symptoms of mental disorders, we look forward to further development of existing models.

## Figures and Tables

**Table 1:** Genes and loci associated with a high risk of developing SZ and other NDDs were investigated with CRISPR/Cas-based genome editors.

Gene/SNP	Genome Editor/Genetic Modification	Molecular/Phenotypic Changes	References
*ARC*	CRISPR/Cas9/gene knockout	Differentially expressed genes and proteins are linked to the extracellular matrix and synapse function	[37]
rs2270363 at 16p13.3	CRISPR/Cas9/deletion in HEK-293T, SH-SY5Y, SK-N-SH, mouse NSCs and rat primary cortical neurons	SNP decreases the expression of *NMRAL1*, which leads to impaired proliferation and differentiation of NSCs and significantly reduces the density of dendritic spikes on neurons.	[38]
rs796364 and rs281759 at 2q33.1	CRISPR/Cas9/deletion in HEK-293T, SH-SY5Y, SK-N-SH, mouse NSCs and rat primary cortical neurons	SNPs involved in the regulation of *TYW5*. Deregulation of *TYW5* leads to defects in NSC proliferation and differentiation as well as dendritic spin density in neurons	[39]
*FOXP2*	CRISPR/Cas9/deletion two downstream enhancers in SK-N-MC and HEK293	Deletion of either of the two enhancers reduced expression of *FOXP2* and its targets in SK-N-MC, but no significant changes were observed in HEK293.	[40]
*DLG2*	CRISPR/Cas9/gene knockout in hESCs	*DGL2* deletion causes deregulation of several transcriptional programs, resulting in delayed neurogenesis, abnormal morphology, migration and action potential generation of differentiated cortical neurons	[41]
*CUL9*	CRISPR/Cas9/single-nucleotide insertions in *CUL9* leading to frame shift mutations	Deletion or depletion of the CUL9 protein in hiPSCs causes aberrant neuronal rosette formation in an in vitro model of early neuralization	[42]
*FURIN*	CRISPR/Cas9/allelic conversion from rs4702 AA to GG	*NGN2*-induced neurons carrying rs4702 GG showed significantly shorter neurite length and significantly shorter average burst duration compared to isogenic controls	[12]
*GRIN2A*	CRISPR/Cas9/A>G nucleotide mutation leading to S644G substitution	Homozygous and heterozygous mutant mice exhibited altered hippocampal morphology at 2 weeks of age, and all homozygotes exhibited lethal tonic-clonic seizures by mid-third week. Heterozygous adults exhibited susceptibility to induced generalized seizures, hyperactivity, repetitive and reduced anxious behavior.	[43]
*Gria3*	CRISPR/Cas9/an orthologous mutation A647T in the mouse *Gria3* gene	The mutation results in an occlusion of the pore in the channel and a deficit in the activation of the corresponding ionotropic glutamate receptor. The mutant mice exhibited slight changes in sleep and activity patterns, as well as increased sensitivity to constant light.	[44]
*KANSL1*	CRISPR/Cas9/a frameshift mutation leading to a premature stop codon in exon 2	*KANSL1* (KAT8 Regulatory NSL Complex Subunit 1) deficiency is associated with increased oxidative stress and autophagy in iPSCs and iNeurons, resulting in reduced synaptic connectivity and neuronal activity both at the individual cell and network level. The observed neuronal phenotype can be restored by treatment with the antioxidant apocynin.	[45]
*SETD1A*	CRISPR/Cas9/heterozygous indels in exon 7 in iPSC-derived glutamatergic/GABAergic neuronal cultures	*SETD1A* loss-of-function mutations result in a morphological increase in dendrite complexity and a functional increase in bursting activity.	[46]
*Stag1* and *Stag2*	CRISPR/Cas9/*Stag1* or *Stag2* gene knockouts in mESC	Inactivation of each of these genes causes a severe depletion of cohesin in chromatin, followed by widespread transcriptome dysregulation and reduced cell proliferation.	[47]
*Bdnf*	CRISPR/Cas9/intronic enhancer deletion in mESC	Strongly reduces both basal and stimulus-dependent levels of exon I-, IIc- and III-containing transcripts of *Bdnf*	[48]
*FMR1*	CRISPR/Cas9/gene knockout	*FMR1*-KO organoids show increased size and number of astrocytes	[49]
*AUTS2* (Autism Susceptibility Gene 2)	CRISPR/Cas9/correction of the de novo missense mutation c.1600 A>C	Restoration of NPCs proliferative activity and cerebral organoid growth	[50]

**Table 2:** CRISPR/Cas9 epigenetic editors are used to study and correct abnormal expression of SZ and other NDDs risk genes.

Target	Epigenome Editor/Model	Observations	References
*PTEN*	CRISPR/dCas-KRAB/9HEK293T, hIPSC-derived neurons	Efficient *PTEN* repression causes increased lengths of neurites	[109]
*GRM2*, *Tent5b, Fos, Sstr2* and *Gadd45b*	SVI-DIO-dCas9-VPR, SVI-DIO-dCas9-KRAB-MeCP2/HEK293, rat primary neurons	Improved CRE-dependent CRISPR activator targeting with no leaky gene induction	[110]
*BDNF*	dCas9-KRAB or VP64-dCas9-VP64/rat primary cortical astrocytes and neurons	Novel intronic enhancer controlling the expression of neuron-specific *Bdnf* transcripts was identified	[48]
*BDNF*	dCas9-KRAB-MeCP2/primary rat hippocampal neuron culture	An improved epigenetic editor provides transcript-selective suppression of *Bdnf* at near-knockout levels	[111]
*SCN1A*	dCas9-VP160/primary hippocampal neurons	Upregulation of the *SCN1A* gene and subsequent increase in Nav1.1 protein level in primary Dravet neurons, which is sufficient to restore the firing rate of GAD67+ GABAergic Dravet interneurons	[112]
*TSNARE1* and *SNAP91*	dCas9-VPR and dCas9-KRAB/NGN2-induced neurons	*SNAP91* and *TSNARE1* deregulation leads to a reduction in synaptic puncta number and size and reciprocal changes in spontaneous excitatory postsynaptic currents	[12]
*FMR1*	dCas9-Tet1/FXS patient-derived iPSCs, neurons	Epigenetic editing activated *FMR1* expression and reversed spontaneous hyperactivity associated with FXS neurons	[113]
*PAX6, ARX*	dCas9-DNMT3A/hESC with *DNMT3A* knockout	Restoration of the differentiation trajectory of *DNMT3A* knockout hESCs: rescue of motor neurogenesis and suppression of floor plate induction.	[114]
*ATP6V1A*	dCas9-KRAB/hiPSC-derived NGN2-induced glutamatergic neurons	Neurons with suppressed *ATP6V1A* show a significant decrease in the number of SYN1+ punctures, neuronal activity, expression of various volt-generated subunits of sodium channels (e.g., *SCN3A*, *SCN2A* and *SCN4B*), the number of full action potentials and an increase in immature spikes.	[115]
*KCTD13, TAOK2, NRXN1, SNAP91, CLCN3*	dCas9-KRAB, dCas9-VP64 and dCas9-VPR/hiPSC-derived NPCs, neurons, and astrocytes	The authors characterized the discrepancies and difficulties in the application of epigenetic CRISPR tools in different cell types	[116]
*Syt1*	dCas9-KRAB/cultured hippocampal neurons, glutamatergic and GABAergic neurons in the dentate gyrus of the mouse hippocampus	Conditional inactivation of *Syt1* shifts the excitation-inhibition (E-I) balance in the dentate gyrus. Shifting the E-I balance toward excitation improved the animals′ ability to spatial distinction. The learning ability of the animals could be bidirectionally regulated, but the mice always exhibited anxious- and depressive-like behavior.	[117]
*TCF4*	Three-component lentiSAMv2 system containing dCas9-VP64, MS2-P65-HSF1 and sgRNA-MS2/brain cortical organoids	*TCF4* overexpression reverses molecular and phenotypic abnormalities associated with PTHS	[118]

## Data Availability

Not applicable.
